# Does depth divide? Variable genetic connectivity patterns among shallow and mesophotic *Montastraea cavernosa* coral populations across the Gulf of Mexico and western Caribbean

**DOI:** 10.1002/ece3.10622

**Published:** 2023-11-08

**Authors:** Alexis B. Sturm, Ryan J. Eckert, Ashley M. Carreiro, Allison M. Klein, Michael S. Studivan, Danielle Dodge Farelli, Nuno Simões, Patricia González‐Díaz, Juliett González Méndez, Joshua D. Voss

**Affiliations:** ^1^ Harbor Branch Oceanographic Institute Florida Atlantic University Fort Pierce Florida USA; ^2^ Rosenstiel School of Marine, Atmospheric, and Earth Science, Cooperative Institute for Marine and Atmospheric Studies (CIMAS) University of Miami Miami Florida USA; ^3^ Atlantic Oceanographic and Meteorological Laboratories (AOML) Miami Florida USA; ^4^ Unidad Multidisciplinaria de Docencia e Investigación–Sisal, Facultad de Ciencias Universidad Nacional Autonoma de México Sisal Yucatán Mexico; ^5^ International Chair for Coastal and Marine Studies, Harte Research Institute for Gulf of Mexico Studies Texas A&M University‐Corpus Christi Corpus Christi Texas USA; ^6^ Laboratorio Nacional de Resiliencia Costera (LANRESC), Laboratorios Nacionales CONACYT Sisal Mexico; ^7^ Centro de Investigaciones Marinas Universidad de La Habana La Habana Cuba; ^8^ Centro Nacional de Áreas Protegidas La Habana Cuba

**Keywords:** 2bRAD, coral refugia, genetic connectivity, Gulf of Mexico, mesophotic coral ecosystems, western Caribbean

## Abstract

Despite general declines in coral reef ecosystems in the tropical western Atlantic, some reefs, including mesophotic reefs (30–150 m), are hypothesized to function as coral refugia due to their relative isolation from anthropogenic stressors. Understanding the connectivity dynamics among these putative refugia and more degraded reefs is critical to develop effective management strategies that promote coral metapopulation persistence and recovery. This study presents a geographically broad assessment of shallow (<30 m) and mesophotic (>30 m) connectivity dynamics of the depth‐generalist coral species *Montastraea cavernosa*. Over 750 coral genets were collected across the Northwest and Southern Gulf of Mexico, Florida, Cuba, and Belize, and ~5000 SNP loci were generated to quantify high‐resolution genetic structure and connectivity among these populations. Generally, shallow and mesophotic populations demonstrated higher connectivity to distant populations within the same depth zone than to adjacent populations across depth zones. However, exceptions to this pattern include the Northwest Gulf of Mexico and the Florida Keys which exhibited relatively high vertical genetic connectivity. Furthermore, estimates of recent gene flow emphasize that mesophotic *M. cavernosa* populations are not significant sources for their local shallow counterparts, except for the Northwest Gulf of Mexico populations. Location‐based differences in vertical connectivity are likely a result of diverse oceanographic and environmental conditions that may drive variation in gene flow and depth‐dependent selection. These results highlight the need to evaluate connectivity dynamics and refugia potential of mesophotic coral species on a population‐by‐population basis and to identify stepping‐stone populations that warrant incorporation in future international management approaches.

## Graphical abstract

We assessed the population genetic connectivity of shallow and mesophotic Montastraea cavernosa corals using a 2bRAD sequencing approach across the Gulf of Mexico and western Caribbean. We found that there was generally significant genetic structuring between shallow and mesophotic depth zones and between the Gulf of Mexico and western Caribbean populations. While the deep reef refugia potential is limited to certain reefs, horizontal connectivity across international boundaries is fairly high in some cases.

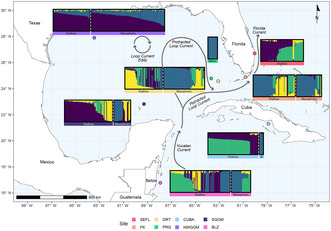

## 1 INTRODUCTION

Over the past half‐century, coral reefs across the Gulf of Mexico and western Caribbean have experienced precipitous declines in coral cover, structural complexity, growth, and overall reef ecosystem health (Alvarez‐Filip et al., 2009; Gardner et al., 2003; Jackson et al., 2014; Perry et al., 2013; Walton et al., 2018). Despite the general trend of significant decline, multiple coral populations within the region have maintained relatively high levels of coral cover, healthy ecosystem services, and may function as potential coral refugia (Kavousi & Keppel, 2018). Understanding the source/sink dynamics among these coral refugia and more highly impacted reefs may facilitate cooperative management approaches to bolster persistence and recovery of regional coral metapopulations (Carson et al., 2011; Jackson et al., 2014).

Of all potential coral refugia, research focus on mesophotic coral ecosystems has increased over recent years largely due to reduced cost and increased safety associated with the remotely operated vehicles and technical diving equipment used to sample and survey these deeper reefs (Armstrong et al., 2019; Pyle, 2019). The deep reef refugia hypothesis (Glynn, 1996) posits that these deep reefs function as coral refugia because their depth and often distance from shore may buffer them from direct human impacts and thermal stress events that regularly affect their shallow reef counterparts. Evidence to support mesophotic refugia capacity is mixed, as there have been observations of mesophotic coral ecosystems experiencing coral bleaching and disease outbreaks and even long‐term datasets identifying significant mesophotic coral cover decline over multi‐decadal scales (de Bakker et al., 2017; Smith et al., 2016; Williams et al., 2021). However, surveys across some mesophotic coral reefs have found relatively low levels of disease and bleaching prevalence, and others have identified long‐term persistence of mesophotic coral communities despite multiple disturbances (Bak et al., 2005; Bloomberg & Holstein, 2021; Hickerson et al., 2012; Reed et al., 2018; Sturm et al., 2021).

While these putative coral refugia may be spatially isolated both from one another and from more highly impacted reefs regionally, strong oceanographic current systems drive larval dispersal and therefore demographic connectivity of corals and other reef‐associated species across geographically discrete reef populations (Roberts, 1997; White et al., 2010). Management of regional coral metapopulations can be improved by characterizing coral source/sink dynamics and quantifying connectivity among coral populations, especially between coral refugia and connected populations downstream (Botsford et al., 2009). These data may then be leveraged to develop optimized networks of marine protected areas or to target highly connected coral populations for focused restoration efforts (Palumbi, 2003). Protecting important coral source populations and their connectivity pathways to promote gene flow toward population sinks can help to maintain high levels of genetic diversity and promote resilience to thermal stress events and disease outbreaks (Van Oppen & Gates, 2006). Collaborative and holistic management of connected coral populations can be challenging as connectivity patterns are poorly understood and can extend across international boundaries, therein requiring cooperation across multiple management and policy frameworks (Nash & McLaughlin, 2014; Strongin et al., 2022).

Regional coral connectivity dynamics are primarily characterized through two approaches: biophysical modeling of larval dispersal and population genetic tools to quantify genetic diversity and differentiation used to infer population connectivity (Botsford et al., 2009; Garavelli et al., 2018; Studivan & Voss, 2018a). Widescale coral biophysical modeling and/or population genetic studies have been conducted for multiple important reef‐building Atlantic coral species including *Orbicella faveolata*, *O. annularis*, *Acropora cervicornis*, *A. palmata*, and *Montastraea cavernosa* as well as more stress‐tolerant, “weedy” coral species like *Porites astreoides* and *Favia fragum* (Baums et al., 2006; Baums, Johnson, et al., 2010; Baums, Paris, & Chérubin, 2010; Devlin‐Durante & Baums, 2017; Foster et al., 2012; Goodbody‐Gringley et al., 2010, 2012; Nunes et al., 2009; Rippe et al., 2017; Serrano et al., 2014, 2016; Studivan & Voss, 2018b). In addition to horizontal geographic separation, the environmental gradients and spatial separation associated with depth are also significant drivers of population genetic structure across coral reef systems (Rippe et al., 2021; Selkoe et al., 2014). However, only a few studies in the Atlantic have incorporated depth stratification and measures of vertical genetic connectivity into their assessment of regional coral metapopulation dynamics, and these studies have found that population genetic evidence for the deep reef refugia hypothesis is mixed (Bongaerts et al., 2017; Brazeau et al., 2013; Liu et al., 2018; Riquet et al., 2022; Serrano et al., 2014, 2016; Studivan & Voss, 2018b; Sturm et al., 2021). Overall, these studies highlight the complexity and variation in regional coral connectivity patterns among coral species with different life history characteristics and ecological niches.

As demonstrated by the many population genetic studies on it *Montastraea cavernosa* is an important coral species for these studies because it is a dominant species in the tropical western Atlantic (Budd et al., 2012; Moyer et al., 2003). The relatively high abundance of this species contributes to its importance as a reef‐builder and facilitates the collection of sufficient sample replicates across multiple reef locations (Horta‐Puga, 2003; Principe et al., 2021). *Montastraea cavernosa* is also an extreme depth generalist found from 1 m down to a record depth of 136 m, making it important to quantify not only regional horizontal connectivity among shallow reefs but also vertical connectivity among shallow and mesophotic depth zones as well (Frade et al., 2019; Reed, 1985). *Montastraea cavernosa* is a gonochoric, broadcast spawning species and while its pelagic larval duration has yet to be quantified in situ, larvae maintained in aquaria survived on average 15 days, suggesting that this species has the potential for high connectivity across large geographic distances (Frys et al., 2020; Kuba, 2016). Despite its ubiquity across the tropical western Atlantic region, *M. cavernosa* is not immune to common coral threats including storm events, bleaching, and disease (Bloomberg & Holstein, 2021; Colella et al., 2012). More recently, the outbreak of stony coral tissue loss disease has led to significant declines in *M. cavernosa* live tissue area and colony density (Alvarez‐Filip et al., 2019; Walton et al., 2018). An understanding of *M. cavernosa* regional genetic connectivity and natural populations' genetic diversity levels could inform restoration and resilience management approaches.

The earliest studies of *M. cavernosa* population genetic structure have primarily assessed horizontal connectivity among relatively shallow sites across multiple spatial scales (Budd et al., 2012; Dodge et al., 2020; Goodbody‐Gringley et al., 2012; Nunes et al., 2011; Serrano et al., 2014). Some studies have also incorporated a depth gradient component. *Montastraea cavernosa*'s prevalence across depth and its wider availability of molecular resources have made it a popular species to assess the deep reef refugia hypothesis from a population genetics standpoint (Brazeau et al., 2013; Eckert et al., 2019; Studivan & Voss, 2018b). However, the majority of these studies used small sets of mitochondrial, microsatellite, AFLP, or other nuclear markers which generally have less power to resolve fine‐scale patterns of population genetic structure as compared to newer approaches, such as Restriction‐site Associated DNA (RAD) sequencing which can produce thousands of single nucleotide polymorphism (SNP) markers (Reitzel et al., 2013). Using datasets with orders of magnitude more genetic markers can lead to more accurate estimates of parameters like fixation index (*F*_ST_) and migration, especially when the number of sample replicates collected for each population are low, which helps with the logistical challenges associated with mesophotic sample collection (Sturm et al., 2020). Only recently have studies involving *M. cavernosa* population genetics began to utilize suites of thousands of SNP markers but often analyzing population genetic structure across smaller geographic scales (Drury et al., 2020; Rippe et al., 2021; Sturm et al., 2020, 2021, 2022). This study builds upon the previous assessments of *M. cavernosa* population genetic connectivity in the Atlantic. A high‐resolution 2bRAD sequencing approach was used to generate thousands of SNP loci to genotype *M. cavernosa* samples collected from shallow and mesophotic reefs across a much wider geographic scale including samples from Florida, the Northwest Gulf of Mexico, the Southern Gulf of Mexico, Cuba, and Belize. This study represents the largest to date of *M. cavernosa* population genetic structure in terms of depth range, number of reefs and populations sampled, number of genetic markers generated, and the total number of unique genets collected providing the most holistic assessment of the connectivity dynamics of this critical coral regional metapopulation.

## 2 MATERIALS AND METHODS

### 2.1 Sample collection and preservation

From 2010 to 2020, 752 unique genets of *M. cavernosa* were collected from reefs across Florida, the Northwest Gulf of Mexico, the Southern Gulf of Mexico, Cuba, and Belize (Table 1). The majority of these samples were collected for earlier, finer‐scale analyses of population genetic structure at each of these reefs (Dodge et al., 2020; Eckert et al., 2019; Studivan & Voss, 2018a, 2018b; Sturm et al., 2020, 2021, 2022). The tissue samples ranged from ~2 to 15 cm^2^ depending on sampling method and the types of additional downstream analyses. *Montastraea cavernosa* were collected from shallow reefs (2–29 m) by either snorkelers or SCUBA divers using a hammer and chisel. Mesophotic samples (30–75 m) were collected by either technical divers on open‐circuit trimix or closed‐circuit rebreathers using hammer and chisels, or by the ROV *Mohawk* employing a five‐function manipulator and suction sampler. Samples were preserved in either TRIzol reagent or 100% molecular‐grade ethanol and were frozen at −20°C until transported back to Harbor Branch Oceanographic Institute where they were kept at −80°C for long‐term storage. In cases where samples had been previously analyzed using microsatellites, care was taken to avoid using multiple samples from previously identified clonal groups to avoid redundant sequencing (Sturm et al., 2020).

**TABLE 1:** 752 unique *Montastraea cavernosa* genotypes were analyzed from eight sites and in most cases across both the shallow and mesophotic depth zones.

Site	Acronym	Depth zone	Reef locations	Depth range (m)	Average depth (m)	*n*_g_	Lat	Long	Source publications
**Southeast Florida**	**SEFL**	**Shallow**		**4.3–22.6**	**13.1**	**66**			Dodge et al. (2020); unpublished data
			St. Lucie Reef‐Central			7	27.13	−80.13	
			St. Lucie Reef‐South			1	27.13	−80.13	
			St. Lucie Reef‐Ledge			6	27.12	−80.13	
			Jupiter			12	26.94	−80.02	
			West Palm			14	26.69	−80.02	
			Boynton			15	26.52	−80.03	
			Fort Lauderdale			11	26.15	−80.10	
**Florida Keys**	**FK**	**Shallow**		**16.8–28.4**	**20.8**	**62**			Sturm et al. (2021)
			Upper Keys‐Carysfort			18	25.22	−80.20	
			Upper Keys‐Elbow			14	25.14	−80.25	
			Lower Keys‐Big Coppitt			29	24.49	−81.60	
			Lower Keys‐Big Coppitt			1	24.49	−81.61	
**Florida Keys**	**FK**	**Mesophotic**		**30.2–45.1**	**37.4**	**38**			
			Upper Keys‐Carysfort			10	25.22	−80.19	
			Upper Keys‐Elbow			7	25.16	−80.22	
			Upper Keys‐Elbow			8	25.15	−80.23	
			Lower Keys‐Big Coppitt			13	24.49	−81.59	
**Dry Tortugas**	**DRT**	**Shallow**		**12.1–27.8**	**21.2**	**55**			Sturm et al. (2021)
			Northern Dry Tortugas‐Sherwood Forest			6	24.68	−83.07	
			Northern Dry Tortugas‐Sherwood Forest			21	24.66	−83.08	
			Southern Dry Tortugas‐Riley's Hump			24	24.52	−83.10	
			Southern Dry Tortugas‐Riley's Hump			4	24.51	−83.10	
**Dry Tortugas**	**DRT**	**Mesophotic**		**29.6–44.8**	**34.6**	**60**			
			Northern Dry Tortugas‐Sherwood Forest			10	24.65	−83.10	
			Northern Dry Tortugas‐Sherwood Forest			13	24.64	−83.10	
			Southern Dry Tortugas‐Riley's Hump			20	24.49	−83.10	
			Southern Dry Tortugas‐Riley's Hump			11	24.49	−83.12	
			Southern Dry Tortugas‐Riley's Hump			6	24.48	−83.11	
**Pulley Ridge**	**PRG**	**Mesophotic**		**62.2–70.4**	**66.4**	**22**			Studivan and Voss (2018b)
			Pulley Ridge			3	24.82	−83.67	
			Pulley Ridge			1	24.81	−83.68	
			Pulley Ridge			2	24.80	−83.67	
			Pulley Ridge			9	24.79	−83.67	
			Pulley Ridge			1	24.78	−83.68	
			Pulley Ridge			2	24.75	−83.70	
			Pulley Ridge			2	24.73	−83.70	
			Pulley Ridge			1	24.71	−83.69	
			Pulley Ridge			1	24.71	−83.70	
**Cuba**	**CUBA**	**Shallow**		**1.5–4**	**3.7**	**77**			Sturm et al. (2020)
			Cayo Jutias			8	22.98	−79.81	
			Guanahacabibes			12	21.80	−84.52	
			Cayo Sabinal			12	21.68	−77.17	
			Isla de la Juventud			3	21.63	−83.21	
			Cabo Lucrecia			16	21.08	−75.64	
			Cayo Anclitas			15	20.81	−78.96	
			Chivirico			11	19.93	−76.40	
**Cuba**	**CUBA**	**Mesophotic**	**Banco de San Antonio**	**40.5 & 74.8**	**57.7**	**2**	22.02	−85.00	
**Northwest Gulf of Mexico**	**NWGOM**	**Shallow**		**19.5–24.9**	**21.9**	**53**			Studivan and Voss (2018a, 2018b)
			East Flower Garden			26	27.91	−93.60	
			West Flower Garden			18	27.87	−93.82	
			West Flower Garden			9	27.88	−93.82	
**Northwest Gulf of Mexico**	**NWGOM**	**Mesophotic**		**30.2–55.3**	**45.8**	**104**			
			East Flower Garden			18	27.91	−93.60	
			East Flower Garden			3	27.92	−93.60	
			West Flower Garden			5	27.87	−93.82	
			West Flower Garden			22	27.88	−93.82	
			Bright			28	27.89	−93.30	
			McGrail			28	27.96	−92.59	
**Southern Gulf of Mexico**	**SGOM**	**Shallow**		**9–29.4**	**16.9**	**71**			Sturm et al. (2022)
			Bajos del Norte			7	23.28	−88.71	
			Bajos del Norte			22	23.25	−88.71	
			Alacranes			6	22.59	−89.75	
			Alacranes			11	22.55	−89.66	
			Alacranes			9	22.51	−89.80	
			Alacranes			3	22.51	−89.63	
			Alacranes			13	22.40	−89.71	
**Southern Gulf of Mexico**	**SGOM**	**Mesophotic**		**33.9–39.3**	**36.5**	**26**			
			Bajos del Norte			11	23.31	−88.72	
			Alacranes			7	22.59	−89.75	
			Alacranes			8	22.40	−89.71	
**Belize**	**BLZ**	**Shallow**		**8.5–29.3**	**17.3**	**87**			Studivan and Voss (2018b); Eckert et al. (2019)
			Tobacco Cay			16	16.83	−88.07	
			South Reef			14	16.77	−88.07	
			Raph's Wall			14	16.78	−88.07	
			Glover's Reef			43	16.76	−87.78	
		**Mesophotic**		**31.4–37.8**	**34.5**	**29**			
			Tobacco Cay			4	16.83	−88.07	
			South Reef			6	16.77	−88.07	
			Raph's Wall			4	16.78	−88.07	
			Glover's Reef			15	16.76	−87.78	

*Note*: The “source publications” indicate the relevant studies these samples were initially used for and provide references for greater details regarding their collection and previous studies of these populations' genetic structure at smaller spatial scales.

Bolding was to highlight and distinguish the regional population level data from the individual site level data.

### 2.2 DNA extraction and 2bRAD library preparation

Tissue from 1 to 2 polyps was scraped from each sample using a sterile scalpel blade. Tissue from samples preserved in TRIzol was transferred directly into a tube with ~0.075 grams of 0.5‐mm glass beads and buffer for extraction. Tissue that was preserved in 100% molecular‐grade ethanol was briefly soaked in TRIzol reagent at 4°C between 1 h and up to multiple days; soaking the tissue in TRIzol reagent improved downstream amplification success. All samples were extracted using a dispersion buffer and phenol:chloroform:isoamyl alcohol separation (see Sturm, 2020 for the detailed protocol) except for the samples collected from Cuba which were extracted using a similar protocol that instead employed a CTAB‐based extraction buffer and a chloroform:isoamyl alcohol phase separation (see Eckert, 2020 for the detailed protocol). All extracted DNA was purified and concentrated using a Zymo DNA Clean and Concentrator kit following the manufacturer's protocols. Extraction quality and purity was measured using a NanoDrop Spectrophotometer and dsDNA quantity was quantified using a Qubit Broad‐Range kit. Sample concentrations were standardized prior to library preparation.

2bRAD libraries were prepared following the protocols detailed by Wang et al. (2012) and the associated GitHub repository (https://github.com/z0on/2bRAD_denovo). Briefly, 100 ng of high‐quality (260/280 values >1.8) DNA was digested with the restriction endonuclease *BcgI* (except samples from Cuba where 200 ng of DNA was incorporated into the digestion reaction). Using the smaller amount of DNA template (100 ng) was still enough DNA to yield successful digestions, and when both amounts were compared, the 100‐ng reactions typically digested and ligated more successfully. This may be because the higher amount of DNA overwhelmed the enzyme or because further diluting the template may have reduced the amount of potential coextracted inhibitors in the digestion reaction. Following digestion, these 2bRAD sequences were prepared as detailed in Sturm et al. (2022, 2021, 2020). Samples for this project were sequenced across multiple single‐end 100 bp sequencing runs on an Illumina NovaSeq with S1 chemistry and 20% *phiX* spike‐in. For each run 4 M reads were targeted for each sample library.

Within each sequencing run, three sample libraries were prepared and sequenced in triplicate. These sample replicates served in the identification of naturally occurring genetic clonal groups and as sequencing quality checks. To ensure that changes in extraction protocol, initial DNA input amount, or sequencing run had no significant effect on downstream genotyping, a subset of replicate 2bRAD libraries were prepared from DNA extracted using both protocols and sequenced across multiple runs. All replicate libraries clustered together as clonal groups, providing evidence that the sequencing data and downstream genotyping are not significantly affected by variation in extraction protocol, initial DNA input amount, or sequencing run biases.

### 2.3 2bRAD bioinformatic analysis

2bRAD reads were demultiplexed and the adapters trimmed using custom Perl scripts (https://github.com/z0on/2bRAD_denovo) and further quality‐filtered using the fastq_quality_filter in the FASTX‐Toolkit (≥90% of bases with Phred quality scores ≥20; Hannon, 2010). An iterative alignment approach divided reads into those that aligned solely to the Symbiodiniaceae genomes and those that aligned solely to the *M. cavernosa* genome. Trimmed and quality‐filtered reads were first aligned to an algal symbiont metagenome assembled by concatenating available genomes for species belonging to four Symbiodiniaceae genera (formerly clades A–D), *Symbiodinium microadriacticum* (Aranda et al., 2016), *Breviolum minutum* (Shoguchi et al., 2013), *Cladocopium goreaui* (Liu et al., 2018), and *Durusdinium trenchii* (Shoguchi et al., 2021) using the sequence aligner Bowtie2 (Langmead & Salzberg, 2012). Reads that aligned to the algal symbiont reference were then aligned to the *M. cavernosa* genome; any reads that aligned to both the Symbiodiniaceae and the coral host genomes (Rippe et al., 2021) were removed from further analysis. Counts of the algal symbiont alignments to each of the four Symbiodiniaceae genera served as a proxy for algal symbiont community structure.

All high‐quality reads that did not align to the algal symbiont metagenomic reference were aligned to the *M. cavernosa* genome. Alignments to the *M. cavernosa* genome were retained for downstream analyses of coral population genetic structure. The program ANGSD was used to generate genotype likelihoods for the dataset and was run with the following filters: a minimum mapping quality score of 20, minimum base quality score of 25, maximum *p*‐value of 10^−5^ that a locus is variable, at least 75% of nonmissing genotypes across samples, minimum *p*‐value for deviation from Hardy–Weinberg equilibrium of 10^−5^, minimum *p*‐value for strand bias of 10^−5^, minimum allele frequency of 0.05, and a filter that removed any tri‐allelic SNPs (Korneliussen et al., 2014). An identity‐by‐state (IBS) matrix was generated for the full dataset and used to create a cluster dendrogram using the function *hclust* in R (R Core Team, 2019). Pairs of samples that exhibited levels of genetic similarity to one another near the similarity level of the technical triplicate groups were identified as naturally occurring genetic clones and omitted from further analyses. ANGSD was rerun on the clones‐removed dataset with the same filters as described above to generate genotype likelihoods and various output files used in downstream analyses including an IBS genetic distance matrix and BCF. In addition, the BCF file generated by ANGSD was converted to a genlight file format suitable for analysis using the program *poppr* in R (Kamvar et al., 2014). *poppr* was used to conduct an analysis of molecular variance (AMOVA, 99 permutations), and the package *StAMPP* was used to calculate pairwise *F*_ST_ between the populations and corresponding *p*‐values (99 permutations; Pembleton et al., 2013).

Ecologically relevant environmental parameters were extrapolated from marine raster layers accessed through BIO‐ORACLE using our sites' geographic coordinates (Assis et al., 2018). While BIO‐ORACLE has both raster layers for the sea surface and “benthic” raster layers extrapolated for the minimum, mean, or maximum depth at a given geographic location's cell, the lack of high‐resolution bathymetry data led to less reliable outputs in certain locations for the benthic layers. Therefore, only values from the sea‐surface layers were retained. The parameters that were downloaded for the assessment included: mean calcite concentration (mol m^−3^), mean percent cloud cover, mean light attenuation coefficient (m^−1^ at 490 nm), mean photosynthetically available radiation (PAR, Einstein m^−1^ day^−1^), mean carbon phytoplankton biomass (μmol m^−3^), mean chlorophyll concentration (mg m^−3^), mean current velocity (m s^−1^), mean dissolved oxygen concentration (μmol m^−3^), mean iron concentration (μmol m^−3^), mean nitrate concentration (μmol m^−3^), mean phosphate concentration (μmol m^−3^), mean primary production (g m^−3^ day^−1^), mean salinity (PSS), mean silicate concentration (μmol m^−3^), and mean sea surface temperature (°C).

In addition, a geographic distance matrix was computed using the *haversine* formula from the *codep* package to compute pairwise distances between sampling locations' geographic coordinates (Guenard et al., 2022). From this distance matrix, distance‐based Moran's eigenvector maps (MEMs) were computed using the package *adespatial* to determine how spatial structure may explain patterns of genetic structure (Dray et al., 2018). MEMs are orthogonal variables that represent the spatial relationships among geographic locations as a large number of eigenfunctions, which can be used as explanatory variables representing spatial structure across multiple scales. Only eigenvalues with significant positive spatial structure (i.e., are spatially autocorrelated) were used in further analyses. The smaller the MEM number (e.g., MEM1), the broader the spatial scale it represents.

These spatial and environmental variables were assessed for collinearity, and when two variables had a correlation coefficient *r* > |.7|, the variable deemed less ecologically relevant was removed. All variables that had a variance inflation factor of >10, indicative of strong multicollinearity, were also removed. Forward, automated, stepwise model selection was employed using the function *ordiR2step* in *vegan* to evaluate the ideal variables to retain in the distance‐based redundancy analysis (dbRDA) model based on adjusted *R* ^2^ and *p*‐values (Oksanen et al., 2019).

Population structure models for clusters *K* = 1–17 (number of populations +3 to identify potentially cryptic genetic structure) were generated using the program NGSAdmix (Skotte et al., 2013). The most likely number of genetic clusters (the best value of *K*) was estimated from likelihood values from each NGSAdmix run (10 iterations for each value of *K*) which were imported into the program StructureSelector, which employs the Puechmaille method to generate four different estimators for the optimal value of *K* (Li & Liu, 2018; Puechmaille, 2016).

Estimates of contemporary migration rates (within the last few generations) among *M. cavernosa* populations were made using the program BA3‐SNPs, a version of the Bayesian modeling program BayesAss3, updated to handle large, SNP datasets (Mussmann et al., 2019; Wilson & Rannala, 2003). First, the BA3‐SNPs‐autotune program was run using a maximum of 10 runs of 10,000 iterations each with 1000 burn‐in to optimize mixing parameters within the desired acceptance rates (Mussmann et al., 2019). The following mixing parameters were accepted by the autotune program and were used in the full runs: migration rate (*m*) = 0.0750, allele frequency (a) = 0.3250, and inbreeding coefficient (f) = 0.0188. Seven repeated runs using these mixing parameter values were run for 18 M iterations with 9 M burn‐in, and 100 sampling intervals. Individual trace files from independent runs were visualized in the program TRACER to ensure model convergence and that runs were consistent with one another. Bayesian deviances were calculated for each run in *R*, Bayesian deviances were similar across all runs but the run with the lowest deviation was used for further analysis. Estimates of migration rates are calculated from means of the posterior distributions of *m* and the associated 95% highest posterior density intervals are reported.

## 3 RESULTS

Following quality filtering steps and the removal of PCR duplicate sequences, technical replicate libraries, and natural genetic clones, ~1.5 B 2bRAD reads or on average 1.98 M reads per sample were retained. The average alignment rate to the Symbiodiniaceae‐concatenated reference was 8.35%. Of the reads that aligned to the Symbiodiniaceae reference, on average 98.9% aligned to the *Cladocopium* genome. From the high‐quality *M. cavernosa* aligned sequences, a set of 5147 SNPs were identified that met the quality filtering parameters and were retained across a minimum of 75% of the samples.

**FIGURE 1:**
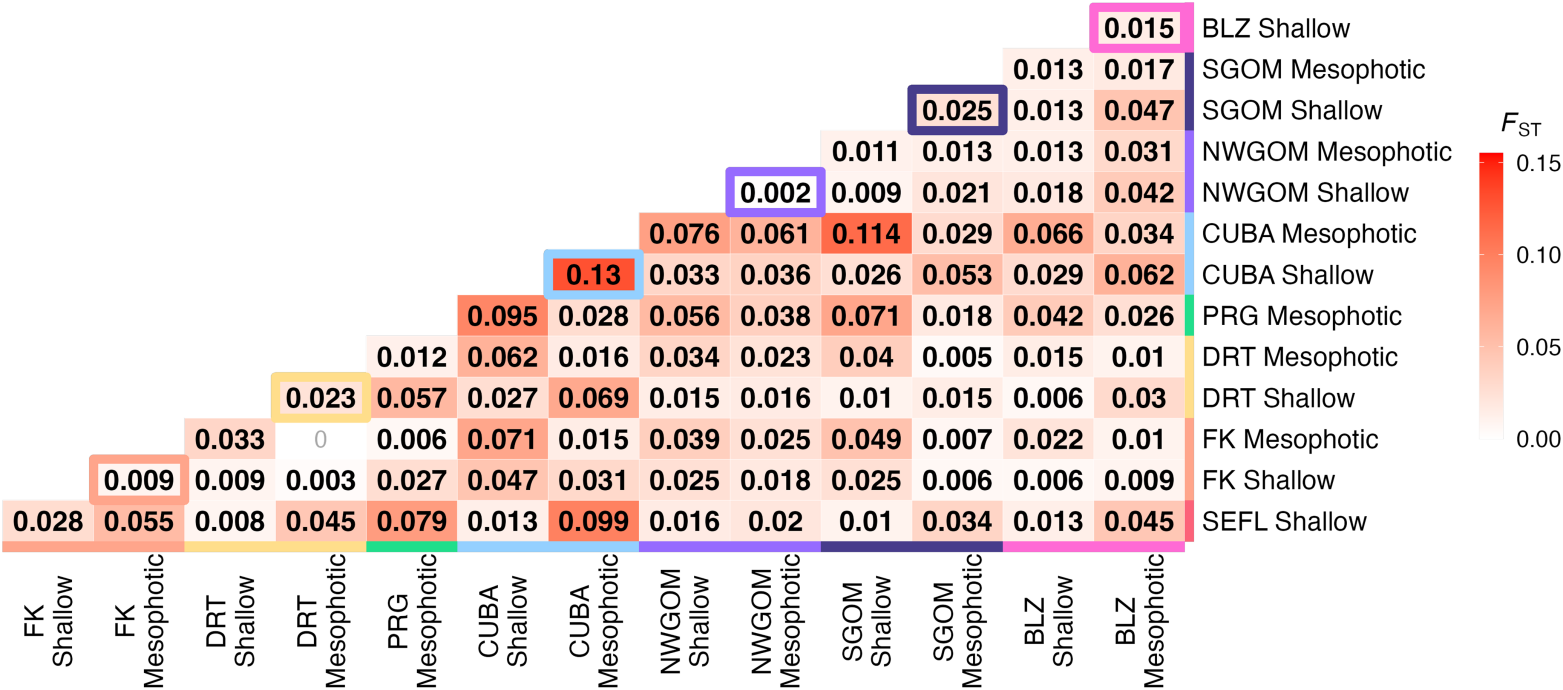
Heat map representations of pairwise population differentiation as estimated by fixation index (*F*_ST_). Values within cells are estimated *F*_ST_ with increasing intensity of the red color corresponding to increasing *F*_ST_ values. Bolded *F*_ST_ values denote significant differentiation between populations (post FDR‐correction, *p* < .05). Outlined boxes are pairwise comparisons of the shallow and mesophotic depth zone at that site indicated by the site's color.

AMOVA attributed a small but significant amount of genetic variation (2.46%, SS = 26,775, *p* < .01) to differences among sample populations. Pairwise *F*_ST_ values were significant for all comparisons except for the adjacent mesophotic Florida Keys and mesophotic Dry Tortugas populations (Figure 1). Pairwise *F*_ST_ values between shallow and mesophotic populations at the same reef varied widely across the region. The pairwise *F*_ST_ between the shallow and mesophotic Northwest Gulf of Mexico populations was near‐zero, *F*_ST_ = 0.002. At the other extreme, the pairwise *F*_ST_ between shallow and mesophotic samples in Cuba was *F*_ST_ = 0.13, the highest pairwise *F*_ST_ value calculated across all population comparisons.

**FIGURE 2:**
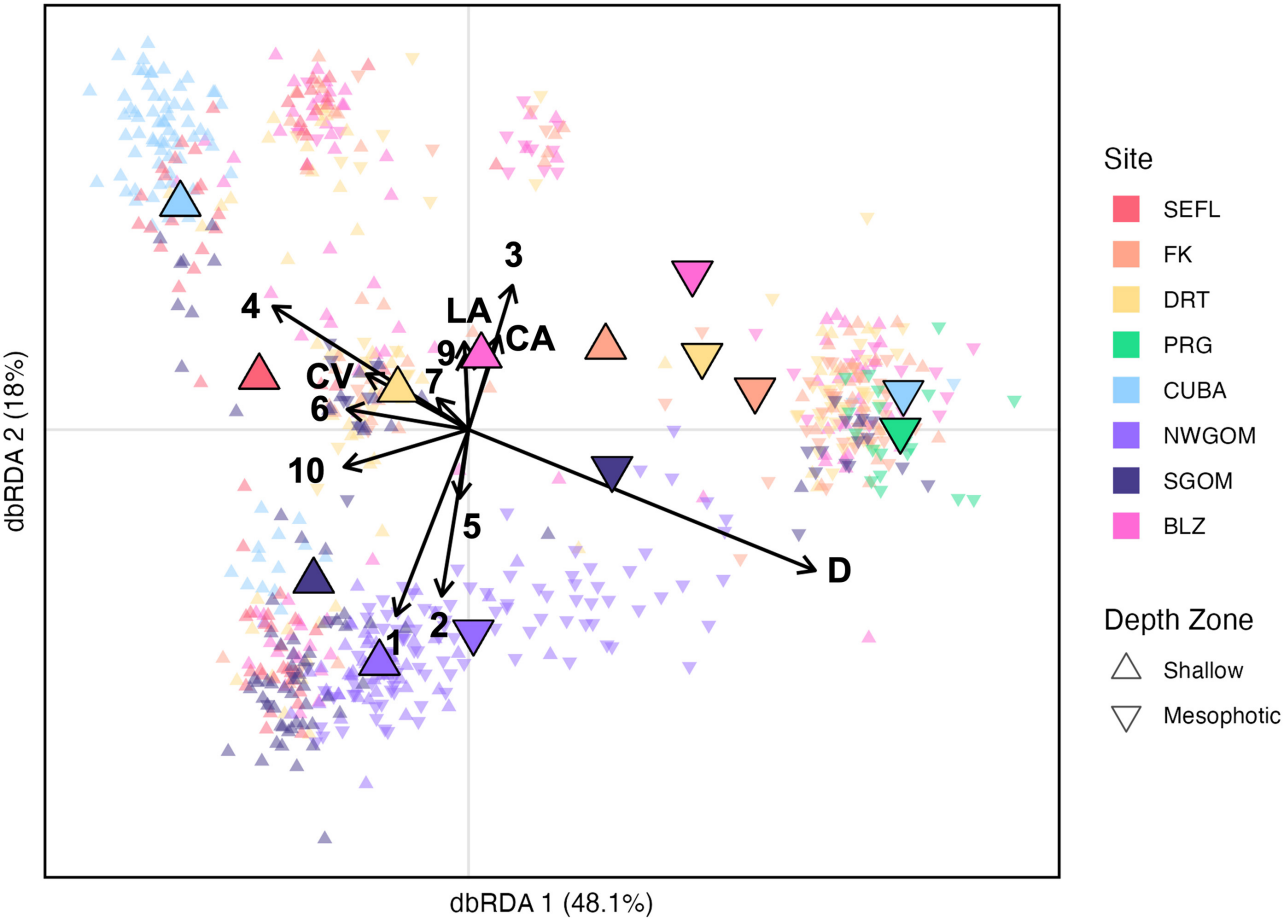
Distance‐based redundancy analysis (dbRDA) of environmental and spatial (Moran's eigenvector maps, MEMs) explanatory variables and genetic response (identity‐by‐state genetic distance matrix). Spatial and environmental variables are represented by the black vectors and their length is proportional to their contribution to each axis, the numbers 1–7, 9–10 represent the retained MEMs; CV, current velocity; LA, light attenuation; CA, calcite, and D, depth. Individual samples are represented by small, transparent symbols whereas population centroids are indicated by larger, solid symbols. Color indicates site and shape indicates depth zone of each sample and population centroid. The percent fitted variation explained by each axis is indicated.

For the dbRDA, after the removal of collinear variables and the automated model selection, the following environmental parameters were retained in the model: sample depth (m), mean light attenuation rate (m^−1^), mean calcite concentration (mol m^−3^), mean sea surface current velocity (m s^−1^), and positive MEMs 1–7, 9, and 10 (Table 2). This model explained a small but significant proportion of the genetic variation (4.8%, *p* < .001) but the first two axes captured 48.1% and 18.0% of the fitted variation, respectively (Table 2; Figure 2). Samples generally clustered by depth zone along dbRDA1 and by location along dbRDA2 (Figure 2). The centroids of all shallow populations were clustered together and the centroids of all mesophotic populations were clustered together. Along dbRDA2, the samples clustered by longitudinal location, with western populations (within the Gulf of Mexico including the Northwest Gulf of Mexico, Southern Gulf of Mexico, and to a lesser extent, Pulley Ridge) clustered with one another and eastern populations (outside of the Gulf of Mexico including Southeast Florida, Florida Keys, Dry Tortugas, Belize, and Cuba) clustered with one another.

**TABLE 2:** Spatial and environmental variables retained in the dbRDA model.

Model variable	Adjusted *R* ^2^	Df	AIC	*F* statistic	*p*‐Value
Depth (m)	.021	1	−2566.982	16.815	.002
MEM1	.026	1	−2570.505	5.521	.002
MEM2	.033	1	−2574.482	5.969	.002
MEM3	.037	1	−2576.515	4.017	.002
MEM4	.041	1	−2578.617	4.081	.002
Current velocity (m s^−1^)	.043	1	−2579.250	2.613	.002
MEM5	.044	1	−2579.172	1.904	.002
MEM9	.045	1	−2578.883	1.692	.002
MEM10	.045	1	−2578.380	1.479	.002
MEM7	.046	1	−2577.828	1.428	.002
MEM6	.047	1	−2577.272	1.423	.002
Light attenuation (m^−1^)	.047	1	−2576.511	1.219	.024
Calcite (mol m^−3^)	.047	1	−2575.887	1.351	.006
Global	.048	13		3.869	.001

Abbreviations: AIC, Akaike information criterion; df, Degrees of freedom; MEM, Moran's eigenvector map.

**FIGURE 3:**
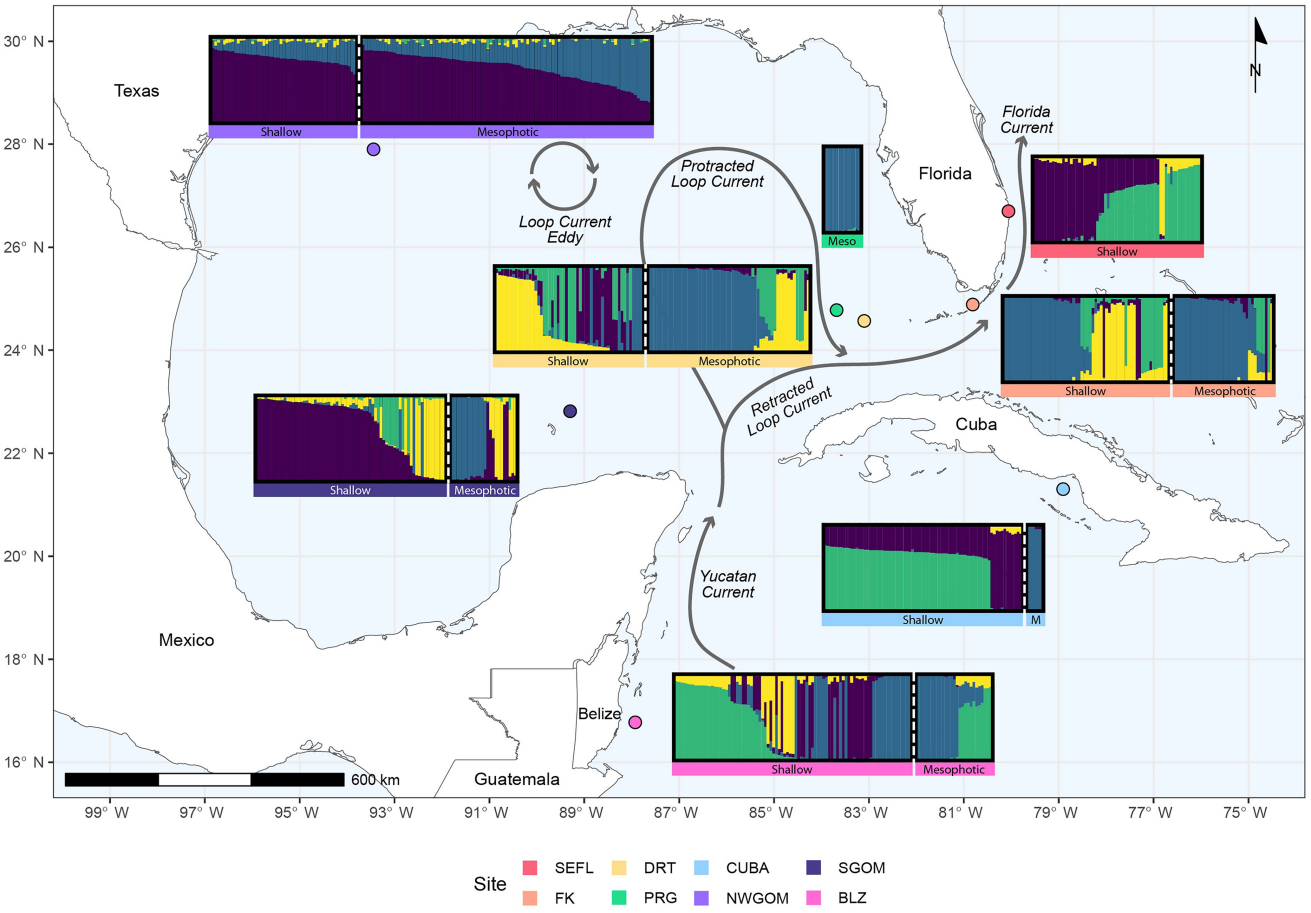
A map of the study region with the centroid of each reef's sampling locations indicated by the colored circles. Overlaid on the map are population structure models generated for the *Montastraea cavernosa* coral populations across each depth zone sampled at each reef. The Puechmaille method was used to estimate the optimal value of *K* and the model *K* = 4 was selected as the most likely number of genetic clusters, represented by the colors green, yellow, purple, and blue. Each panel is proportional to the number of *M. cavernosa* included in that population's analysis, except for the mesophotic Cuba population which only had two sample replicates, therefore, this panel was enlarged to improve visibility. Each bar indicates individual *M. cavernosa* samples and the relative proportion of the four colors represents the relative likelihood of membership with each of the four proposed genetic clusters. Shallow and mesophotic samples are separated by a dotted, white line. Dark gray, stylized arrows represent components of the Loop Current system adapted from NASEM (2018).

Based on StructureSelector model validation and visualization of NGSAdmix outputs, *K* = 4 was chosen as the most likely number of genetic clusters for plotting population genetic structure (Figure 3).We examined admixture plots for *K* = 2–5 to examine how patterns of genetic variation change across different numbers of clusters (*K*; Figure S1). Generally, there was high intrapopulation variability, with many populations hosting samples dominated by multiple different genetic clusters (Figure 3). For example, shallow and mesophotic Florida Keys, shallow Dry Tortugas, shallow Southern Gulf of Mexico, and shallow Belize have samples dominated by each of the four genetic clusters. On the other hand, Pulley Ridge and mesophotic Cuba, the deepest populations are dominated almost exclusively by the blue genetic cluster (although mesophotic Cuba has only two sample replicates).

For reef locations where shallow and mesophotic populations were both sampled, the level of genetic structuring between depth zones varied. In the Northwest Gulf of Mexico, both the shallow and mesophotic populations consisted of samples primarily admixed between the blue and purple genetic clusters (Figure 3). Similarly, shallow and mesophotic populations in the Florida Keys shared a high prevalence of the blue and green genetic cluster, although the yellow genetic cluster was more predominant across the shallow Florida Keys population. Both the shallow and mesophotic Dry Tortugas populations had individuals dominated by the yellow and green genetic clusters. However, the blue genetic cluster was much more prevalent in the mesophotic zone of the Dry Tortugas, while the purple genetic cluster was more prevalent in the shallows. Similarly in the Southern Gulf of Mexico, the yellow genetic cluster was present in both the shallow and mesophotic zones, but the shallow had a much higher prevalence of the purple genetic cluster and the mesophotic had a much higher prevalence of the blue genetic cluster. Both the shallow and mesophotic depth zones in Belize shared samples dominated by the blue and green genetic clusters but some shallow samples in Belize were dominated by or admixed with the purple and yellow genetic clusters which were not found in the mesophotic zone. Shallow samples across Cuba were dominated by either the green or the purple genetic cluster, which made it completely distinct from the mesophotic population which was dominated by the blue genetic cluster.

**FIGURE 4:**
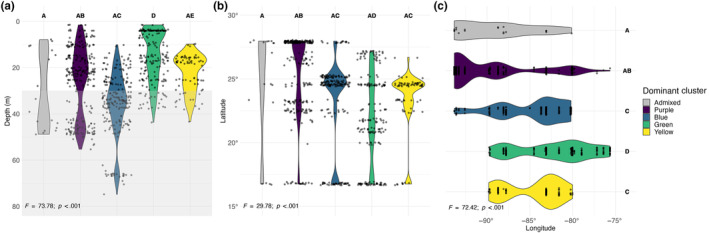
Violin plots depicting the distribution of dominant genetic clusters across (a) depth, (b) latitude, and (c) longitude. If a sample was not dominated by a single cluster (>50% membership to one cluster) then it was considered admixed. The gray overlay on Figure 4a indicates mesophotic depth zones (>30 m). Results of Welch's ANOVA are indicated in the bottom left corner of each plot. Game's Howell post hoc comparisons are indicated with letter annotations.

When plotting samples dominated by each of the four genetic clusters, as well as admixed samples (samples that did not have >50% of a single genetic cluster), across depth, latitude, and longitude, some regional patterns occur (Figure 4). For example, while the blue genetic cluster is found across a shallow to mesophotic depth gradient, it is more prevalent at mesophotic depths (Figure 4a). When examining variation across longitude, we also find that the purple cluster is more prevalent across western sites (i.e., within the Gulf of Mexico), the green genetic cluster is more dominant across eastern sites (i.e., within the western Caribbean), and the blue and yellow genetic clusters are more centrally located (Figure 4c). Similarly, the purple genetic cluster was more prevalent at higher latitudes, the green genetic cluster more prevalent at lower latitudes, and the blue and yellow clusters more prevalent at mid‐latitudes (Figure 4b). Although, the variation of clusters across latitude, while still significant, was much lower than across depth or longitude (Welch's ANOVA; *F*_depth_ = 73.78, *p*_depth_ < .001; *F*_latitude_ = 29.78, *p*_latitude_ < .001; *F*_longitude_ = 72.42, *p*_longitude_ < .001).

**FIGURE 5:**
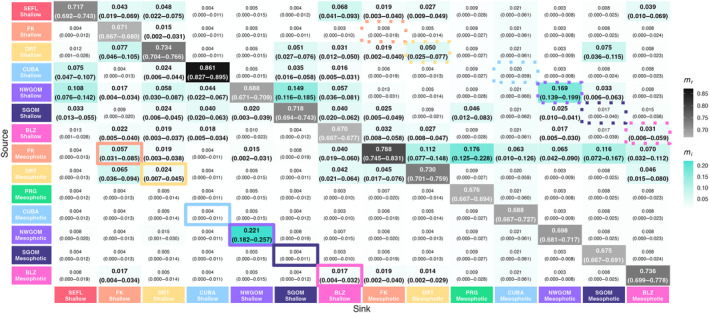
A pairwise migration rate (*m*) heat map displaying mean migration rate and the 95% highest posterior density (HPD) interval surrounding it. Enlarged, bolded migration rates have a HPD that does not contain zero. Source populations are on the *Y* axis and sink populations on the *X* axis and are listed and indicated by color. The higher the immigration (i.e., *m*_*i*_, migrants from one source population retained in a distinct sink population) the more intense the aqua color. Local retention to an individual population (i.e., *m*_*r*_) is indicated in grayscale with darker colors indicating higher retention. Connectivity between depth zones at the same site is outlined by that site's color, with dotted lines indicating shallow to mesophotic gene flow and solid lines indicating mesophotic to shallow gene flow.

Analysis of recent migration found that overall average migration rates were low (mean ± SEM, 2.2 ± 0.2%) and that shallow populations on average were more important sources than mesophotic populations (shallow: 2.5 ± 0.3%, mesophotic: 1.9 ± 0.4%; Table 3). More specifically, shallow to mesophotic subsidy (2 ± 0.4%) was higher than mesophotic to shallow subsidy (1.4 ± 0.5%). However, the largest population source of all pairwise comparisons was the mesophotic Northwest Gulf of Mexico to its shallow counterpart (22.1%); the shallow Northwest Gulf of Mexico population is also a significant source to its mesophotic counterpart (16.9%; Figure 5). The shallow Northwest Gulf of Mexico population is also a significant source to other shallow populations across the region (1.7%–14.9%) while the mesophotic Florida Keys population is an important source for other mesophotic populations in the region (6.3%–17.6%). While the proportion of individuals retained in a certain population was high across all populations, shallow Cuba had the highest proportion of individuals sourced from itself (86.1%).

**TABLE 3:** Mean, standard deviation, and standard error of recent migration rates (*m*) calculated using BA3‐SNPs (Mussmann et al., 2019) for each dataset listed.

Dataset	Mean migration rate (*m*)	Standard deviation	Standard error
Global	0.022	0.032	0.002
Mesophotic source	0.019	0.035	0.004
Shallow source	0.025	0.029	0.003
Mesophotic sink	0.022	0.031	0.003
Shallow sink	0.021	0.033	0.003
Mesophotic ➔ Shallow	0.014	0.033	0.005
Mesophotic ➔ Mesophotic	0.024	0.036	0.006
Shallow ➔ Mesophotic	0.020	0.025	0.004
Shallow ➔ Shallow	0.029	0.031	0.005

## 4 DISCUSSION

### 4.1 Variable patterns of vertical connectivity

In general, shallow *M. cavernosa* populations were more genetically similar to other shallow populations than to their mesophotic counterparts, and vice versa (Figure 1; Figure 2). Additionally, contemporary vertical migration from mesophotic sources to shallow sinks was lower than mesophotic sources to mesophotic sinks and shallow sources to shallow sinks (Table 3). This highlights that, from a genetic connectivity standpoint, there is limited evidence of universally high refugia potential of these mesophotic *M. cavernosa* populations. Vertical connectivity varied highly across sites and there were some examples of high connectivity between shallow and mesophotic depth zones at the same reef. Notably, vertical connectivity was the highest between shallow and mesophotic depth zones in the Northwest Gulf of Mexico (*F*_ST_ = 0.002; Figure 1), reaffirming findings previously identified through microsatellite analyses (Studivan & Voss, 2018a). Estimates of recent migration also highlight transfers among mesophotic and shallow populations in the Northwest Gulf of Mexico (mesophotic to shallow: 22.1%, shallow to mesophotic: 16.9%; Figure 5). The low genetic differentiation between shallow and mesophotic populations in the Northwest Gulf of Mexico may be heavily influenced by eddies shed from the Loop Current which are thought to drive larval retention and cross‐depth zone connectivity among the sampled banks (Garavelli et al., 2018; Limer et al., 2020).

Genetic connectivity was also relatively high between shallow and mesophotic Florida Keys populations (*F*_ST_ = 0.009; Figure 1), and this was especially observed in the Upper Keys, reaffirming the pattern identified by Sturm et al. (2021) on a regional scale. Estimates of more recent migration also demonstrated that the mesophotic Florida Keys population is a significant source to its shallow counterpart (5.7%), but the relationship is not reciprocated. A biophysical modeling study of *M. cavernosa* larval connectivity in the Florida Keys suggested that strong tidal currents in the Upper Keys promote high levels of larval retention (Frys et al., 2020). The potential environmental or biophysical drivers of vertical gene flow are still not well characterized for *M. cavernosa*; however, previous studies have hypothesized that it may be related to environmental shifts driven by reef geomorphology (Eckert et al., 2019; Sturm et al., 2022). In both the Northwest Gulf of Mexico and the Florida Keys, the predominant biophysical regimes like persistent eddies or strong tidal influence both promoted higher levels of local larval retention and may also drive vertical mixing of the water column which may synergistically boost levels of vertical connectivity (Furuichi & Hibiya, 2015).

Of the genetic structure observed in this study, colony depth explained a significant level of genetic variation (Figure 2). Pairwise shallow to mesophotic measures of genetic differentiation in Belize, Dry Tortugas, Southern Gulf of Mexico, and Cuba were relatively high (*F*_ST_ = 0.015–0.13; Figure 1). It is possible that depth functions as a spatial driver of genetic differentiation, that is colonies have a vertical distance separating them. This is especially relevant to a broadcast‐spawning coral like *Montastraea cavernosa*. Their colonies release positively buoyant sperm and eggs into the water column, which break apart and fertilize near the surface (Levitan et al., 2004). Even if corals spawn synchronously across depth, gametes from deeper corals have to traverse a greater distance and therefore it may take longer for them to reach the surface, potentially driving temporal reproductive isolation between shallow and mesophotic coral populations (Vize, 2006). Moreover, at certain sites, oceanographic regimes may vary across depth so it is possible that gametes from shallow and deeper corals may be advected away from one another before they can mix at the surface (Holstein et al., 2015). In addition to a spatial separation component, environmental factors related to depth may also be driving genetic differentiation via adaptation to different environmental niches. Perhaps one of the most important environmental drivers of coral reef community structure across depth is light (Laverick et al., 2020; Lesser et al., 2018). While in situ light levels were not collected in the study, light attenuation was retained in the dbRDA model as a significant driver of genetic structuring (Table 2). Therefore, it is possible that adaptation to varying light regimes across depth contributes to the depth‐dependent genetic differentiation observed.

Previous studies of vertical genetic connectivity in benthic macroinvertebrates are often limited in geographic spatial scale but have identified a variety of structuring patterns across different species. The broadcast spawner, *Stephanocoenia intersepta*, exhibited no genetic structuring between shallow (~12 m) and mesophotic (~40 m) depth zones across sites in Bermuda (Bongaerts et al., 2017). However, samples of a brooding coral, *Agaricia fragilis*, collected at the same sites and depth ranges exhibited significant genetic differentiation between shallow and mesophotic depth zones. Surprisingly, a study of another putative brooding species, *Agaricia undata*, found no genetic structuring along a 17–45 m depth range across sampling sites in Cartagena, Colombia (Gonzalez‐Zapata et al., 2018). Similarly, high connectivity between shallow (10–20 m) and mesophotic (30–40 m) sites in southwestern Puerto Rico was also identified in another brooding coral species, *Agaricia lamarcki* (Hammerman et al., 2018).

Regional Atlantic coral metapopulation dynamics that have incorporated depth‐stratified sampling are rare, but they have similarly identified location‐based variation in the level of vertical connectivity within a species. *Porites astreoides* and *M. cavernosa* sampled across ≤10, 15–20, and ≥25 m depth zones across Florida, the US Virgin Islands, Bermuda, and Guadeloupe (for *P. astreoides* only) populations found high levels of depth‐dependent genetic differentiation across Florida and low levels of differentiation between shallow and deep zones in Bermuda identified for both species, and Guadeloupe for *P. astreoides* (Riquet et al., 2022; Serrano et al., 2014, 2016). In contrast, the level of vertical connectivity in the US Virgin Islands varied between species with high levels of differentiation between shallow and deep populations of *P. astreoides* but not *M. cavernosa*. *Montastraea cavernosa* collected across wide shallow (3–25 m) and mesophotic (30–90 m) depth clines across the Bahamas and Little Cayman Island, over 1000 km away, identified significant genetic differentiation between shallow and mesophotic populations in Little Cayman but not in the Bahamas (Brazeau et al., 2013). An earlier regional analysis of *M. cavernosa* samples collected from shallow (15–30 m) and mesophotic (30–70 m) reefs across southwestern Florida, the Flower Garden Banks, and Belize using microsatellite markers (of which many of these samples were reanalyzed with SNPs) also exhibited variable levels of vertical connectivity across depth based on location. The Flower Garden Banks exhibited low levels of genetic differentiation across depth, as mentioned previously and reaffirmed in this study, while shallow populations in Belize and southwestern Florida were genetically distinct from their mesophotic counterparts, similar to the patterns noted in this study (Studivan & Voss, 2018b). Similarly, the broadcast spawning barrel sponge, *Xestospongia muta*, exhibited no significant genetic structuring between lower mesophotic Pulley Ridge, (61–69 m) and much shallower depth ranges in the Dry Tortugas (27–32 m; Bernard et al., 2019). This is comparable to this study which identified low connectivity between Pulley Ridge (~66 m) and the shallow Dry Tortugas (~21 m) but higher connectivity to the mesophotic Dry Tortugas (~35 m). While not a regional analysis, another study sampled *M. cavernosa* across a shelf gradient from a shallow nearshore patch reef (3–5 m), to a shallow offshore backreef (~10 m), and a deep offshore site (~20 m; Rippe et al., 2021). These samples were genotyped with 2bRAD approaches and similarly they identified significant genetic structuring among the nearshore shallow site, offshore shallow site, and offshore deep site, which they attributed to environmental specialization of the *M. cavernosa* across depth. Overall, this study, like those conducted previously, highlights the variability in vertical genetic connectivity across species and even within a species across reef locations and emphasizes the importance of assessing refugia potential on a species‐by‐species and population‐by‐population basis.

### 4.2 Significant regional genetic structuring between the Gulf of Mexico and western Caribbean sites

In addition to significant levels of genetic structuring across depth, spatial variables (in the form of MEMs) also explained a significant amount of genetic variation (i.e., isolation by distance; Table 2, Figure 2). One of the spatio‐genetic patterns observed in this dataset was the differentiation between the Gulf of Mexico populations: Northwest Gulf of Mexico, Southern Gulf of Mexico, and Pulley Ridge and western Caribbean populations: Belize, Cuba, Dry Tortugas, and Southeast Florida (Figures 3 and 4c). In the dbRDA ordination, these populations tend to separate from one another along dbRDA2 (Figure 2). In the admixture plots, the Northwest Gulf of Mexico populations and the shallow Southern Gulf of Mexico population are dominated by the purple genetic cluster, while western Caribbean populations tended to have a higher frequency of samples belonging to the green genetic cluster (Figure 3), and these two clusters diverge significantly across longitude (Figure 4c).

Regional studies of other coral species focused primarily on the wider Caribbean have identified a semipermeable bio‐oceanographic break associated with eddies that occur within the Mona Passage located between Hispaniola and Puerto Rico, preventing larvae from traversing this area and driving genetic differentiation between the western and eastern Caribbean (Baums, Paris, & Chérubin, 2010; Rippe et al., 2017). The results of this study suggest that the powerful Loop Current system may similarly act as a barrier to genetic connectivity between the Gulf of Mexico and the western Caribbean (Figure 3).

However, the Loop Current system exhibits significant intra‐ and inter‐annual variability in its intrusion and reach into the Gulf of Mexico (Weisberg & Liu, 2017). In years where the current is “protracted” and intrudes deeply into the Gulf, there may be increased interaction between Gulf of Mexico reefs and the Loop Current system which may drive pulses of connectivity from these reefs to reefs in the western Caribbean (Sanvicente‐Añorve et al., 2014). This may be why we observe some members of the putative “Gulf‐dominant” purple genetic cluster across Belize and Cuba and in populations down current, like in Southeast Florida, and why there is evidence of the Northwest Gulf of Mexico acting as an important source to these populations in assessments of recent migration rates (Figures 3 and 5; Sheinbaum et al., 2016; Carrillo et al., 2017). Following instances of increased intrusion in the Gulf of Mexico, eddies often pinch off and travel westward throughout the Gulf (Sheinbaum et al., 2016; Weisberg & Liu, 2017). Larvae from western Caribbean sites could get entrained in these mesoscale eddies that then may move across Gulf reefs. This may drive sporadic larval migration from the western Caribbean, especially the Florida Keys and the Dry Tortugas to the Gulf of Mexico reef populations (Olascoaga et al., 2018).

### 4.3 High connectivity between the southwestern and northwestern Caribbean

While the Loop Current system appears to function as a semipermeable barrier to connectivity between western and eastern sites, its high northerly velocities may be driving connectivity from southwestern Caribbean sites to northwestern Caribbean sites. There were notable levels of genetic connectivity between Belize and the Dry Tortugas, Florida Keys, and southeastern Florida despite geographic separation as high as 1400 km. Studivan and Voss (2018b) identified high cross‐depth zone connectivity between mesophotic Belize and the shallow Dry Tortugas populations, but we identified the highest levels of connectivity across these two regions between populations at the same depth zone. Reported velocities of the Loop Current system range but can reach maximums of 1.2–2 m s^−1^ at the Yucatan and Florida straits, and peaks in mean transport tend to occur during the summer months which coincides with *Montastraea cavernosa*'s spawning season (Candela et al., 2019; Centurioni & Niiler, 2003; Jordan, 2018; Szmant, 1991). Given the high velocity of surface currents during spawning season and the relatively high pelagic larval duration of broadcast‐spawning corals, it would be possible for gene flow to occur from coral larvae originating from Belize and elsewhere on the Mesoamerican reef with successful downstream recruitment on Florida's reefs either through intergenerational stepping stone pathways or even directly (Lugo‐Fernández, 2006). However, estimates of recent migration rates between the two populations actually suggest that there are cases of bidirectional connectivity between the two reef systems. Counterintuitively, southeastern Florida was estimated to be a more significant source to shallow Belize than the reverse scenario (Figure 5). Overall, the intra‐ and inter‐annual variability in the Loop Current system may play a role in the differences observed between historical and more recent gene flow patterns.

### 4.4 *Montastraea cavernosa*'s regional connectivity dynamics: Implications for management

*Montastraea cavernosa* is a ubiquitous, dominant, extreme depth‐generalist broadcast spawning species that is perhaps one of the most well‐studied coral species in the region (Lesser et al., 2018). While it is only one species, it can serve as a model for other ubiquitous reef‐associated species with similar life history patterns. In this study, a greater understanding of regional *M. cavernosa* connectivity dynamics has provided important evidence for the need for more holistic and cooperative coral reef management approaches. In some areas like the Northwest Gulf of Mexico and the Florida Keys, mesophotic *M. cavernosa* populations do appear to meet the underlying assumptions of the deep reef refugia hypothesis (Bongaerts et al., 2010; Glynn, 1996). Both mesophotic populations maintain relatively high levels of coral cover and serve as depth refugia from threats like coral disease outbreaks (Hickerson et al., 2012; Johnston et al., 2016; Reed et al., 2015; Sturm et al., 2021). Additionally, they demonstrate relatively high levels of historical and more recent gene flow to their shallow counterparts, indicative of larval connectivity between them. However, high levels of vertical genetic connectivity are far from universal and *M. cavernosa* are much more likely to be genetically similar to other corals within their same depth zone. The refugia potential of reefs varies widely and as shallow coral reefs (and to a certain extent mesophotic coral reefs as well) face increasingly compounding threats and severe declines, mesophotic populations cannot solely be depended on to reseed shallow populations. This genetic dataset showcases that, in certain areas, mesophotic populations host unique genetic lineages not found prevalently across their shallow counterparts (e.g., the blue genetic cluster across the mesophotic Dry Tortugas, Southern Gulf of Mexico, and Cuba; Figure 3). Therefore, we recommend greater inclusion of mesophotic populations into existing monitoring and management plans and consideration of new approaches tailored to shallow and mesophotic populations in order to maintain the highest level of coral metapopulation genetic diversity.

High levels of *M. cavernosa* genetic similarity across the Gulf of Mexico coral reefs in US, Cuban, and Mexican waters provide evidential support for the need to cooperatively manage these interconnected coral populations. There are already bilateral cooperative management agreements in the form of “Memorandums of Understanding” (MOU; Strongin et al., 2022). The first of which was signed by the United States and Cuba in 2015 developing a “sister sanctuaries” program among coral reef marine protected areas including the Flower Garden Banks National Marine Sanctuary (in the Northwest Gulf of Mexico), Florida Keys National Marine Sanctuary, and Guanahacabibes and Banco de San Antonio marine protected areas in western Cuba (NOAA, 2015). In 2018, a similar MOU was signed by Cuba and Mexico recognizing the need for cooperative marine protected area management (Strongin et al., 2022). However, there is currently no overarching agreement among the United States, Cuba, and Mexico to cooperatively manage the Gulf of Mexico coral ecosystems although multiple tri‐national management frameworks have been proposed (Nash & McLaughlin, 2014; Strongin et al., 2022). Moreover, it should be noted that *M. cavernosa* populations off the coast of Belize demonstrate strong genetic connectivity to coral reef systems in Florida and further investigations should consider employing biophysical modeling techniques to assess the importance of Belize coral populations as potential larval sources to downstream reefs in the Gulf of Mexico and western Caribbean and if these populations warrant incorporation into proposed regional management frameworks. In general, continued effort is needed to expand the diversity of coral species for which we have regional genetic connectivity data across shallow and mesophotic depth zones to provide a more holistic understanding of coral connectivity dynamics and population persistence. With these diverse and wide‐ranging genetic datasets, we can better inform management approaches that support the resiliency of these regional metapopulations, something that will become ever more critical as they experience increasingly severe effects of both localized and global anthropogenic stressors including climate change.

## AUTHOR CONTRIBUTIONS

**Alexis B. Sturm:** Conceptualization (equal); data curation (lead); formal analysis (lead); funding acquisition (supporting); investigation (lead); methodology (equal); project administration (supporting); visualization (equal); writing – original draft (lead). **Ryan J. Eckert:** Conceptualization (supporting); data curation (supporting); formal analysis (equal); investigation (supporting); methodology (supporting); validation (equal); visualization (equal); writing – review and editing (supporting). **Ashley M. Carreiro:** Data curation (supporting); investigation (supporting); writing – review and editing (equal). **Allison M. Klein:** Data curation (supporting); investigation (supporting); writing – review and editing (equal). **Michael S. Studivan:** Conceptualization (supporting); data curation (supporting); investigation (equal); project administration (supporting); resources (equal); writing – review and editing (supporting). **Danielle Dodge Farelli:** Conceptualization (supporting); data curation (supporting); investigation (equal); writing – review and editing (supporting). **Nuno Simões:** Funding acquisition (supporting); project administration (equal); resources (equal); supervision (equal); writing – review and editing (supporting). **Patricia González‐Díaz:** Data curation (supporting); investigation (equal); project administration (equal); supervision (supporting); writing – review and editing (supporting). **Juliett González Méndez:** Data curation (supporting); investigation (supporting); writing – review and editing (supporting). **Joshua D. Voss:** Conceptualization (equal); data curation (supporting); funding acquisition (lead); investigation (supporting); methodology (supporting); project administration (lead); resources (equal); supervision (lead); writing – review and editing (equal).

## CONFLICT OF INTEREST STATEMENT

The authors have no competing interests to disclose.

### OPEN RESEARCH BADGES

This article has earned Open Data and Open Materials badges. Data and materials are available at [[insert provided URL(s) on the Open Research Disclosure Form]].

### DATA AVAILIBILITY STATEMENT

Trimmed, deduplicated, and quality‐filtered 2bRAD sequences and sample metadata are uploaded to the National Center for Biotechnology Information Sequence Read Archive as part of following BioProjects: PRJNA1024506‐Southeast Florida, Pulley Ridge, Northwest Gulf of Mexico, and Belize; PRJNA742926‐Florida Keys and Dry Tortugas; PRJNA626681‐Cuba; and PRJNA789549‐Southern Gulf of Mexico. Associated data files including IBS matrix, BCF, NGSAdmix output files, analysis and figure generation scripts, and protocols are available through the Sturm et al. (2023)‐archived GitHub repository: https://github.com/lexiebsturm/regionalMcavConnectivity.

## Supporting information


Data S1.
