# Supplementary material for: Does depth divide? Variable genetic connectivity patterns among shallow and mesophotic Montastraea cavernosa coral populations across the Gulf of Mexico and western Caribbean
Source: Ecol Evol. 2023 Nov 8;13(11):e10622. doi: 10.1002/ece3.10622 (PMC10631546; doi:10.1002/ece3.10622)
Supplement: Supplementary file 1 — Data S1. [file ECE3-13-e10622-s001.docx]

**TITLE**

Does depth divide? Variable genetic connectivity patterns among shallow and mesophotic *Montastraea cavernosa* coral populations across the Gulf of Mexico and western Caribbean

**RUNNING TITLE**

Variable vertical coral genetic connectivity

**AUTHORS**

Alexis B. Sturm^1*^, Ryan J. Eckert^1^, Ashley M. Carreiro^1^, Allison M. Klein^1^, Michael S. Studivan^1,2,3^, Danielle Dodge Farelli^1^, Nuno Simões^4,5,6^, Patricia González-Díaz^7^, Juliett González Méndez^8^, and Joshua D. Voss^1**^

**AUTHOR AFFILIATIONS**

^1^Harbor Branch Oceanographic Institute, Florida Atlantic University, 5600 US 1 North, Fort Pierce, FL 34946, USA

^2^Rosenstiel School of Marine, Atmospheric, and Earth Science, Cooperative Institute for Marine and Atmospheric Studies (CIMAS), University of Miami, 4600 Rickenbacker Cswy., Miami, FL 33149, USA

^3^Atlantic Oceanographic and Meteorological Laboratories (AOML), NOAA, 4301 Rickenbacker Cswy., Miami, FL 33149, USA

^4^Unidad Multidisciplinaria de Docencia e Investigación–Sisal, Facultad de Ciencias, Universidad Nacional Autonoma de México, Puerto de Abrigo S/N, Sisal, Yucatán, Mexico 97355

^5^International Chair for Coastal and Marine Studies, Harte Research Institute for Gulf of Mexico Studies, Texas A&M University-Corpus Christi, Corpus Christi, TX 78412, USA

^6^Laboratorio Nacional de Resiliencia Costera (LANRESC), Laboratorios Nacionales, CONACYT, Sisal 97356, Mexico

^7^Centro de Investigaciones Marinas, Universidad de La Habana, Calle 16, No. 114, Miramar, La Habana, Cuba

^8^Centro Nacional de Áreas Protegidas, Calle 18a, No. 1441, Playa, La Habana, Cuba

Corresponding Authors’ Contacts: *[asturm2017@fau.edu](mailto:asturm2017@fau.edu); **[jvoss2@fau.edu](mailto:jvoss2@fau.edu)

**Supplementary Figures**

**
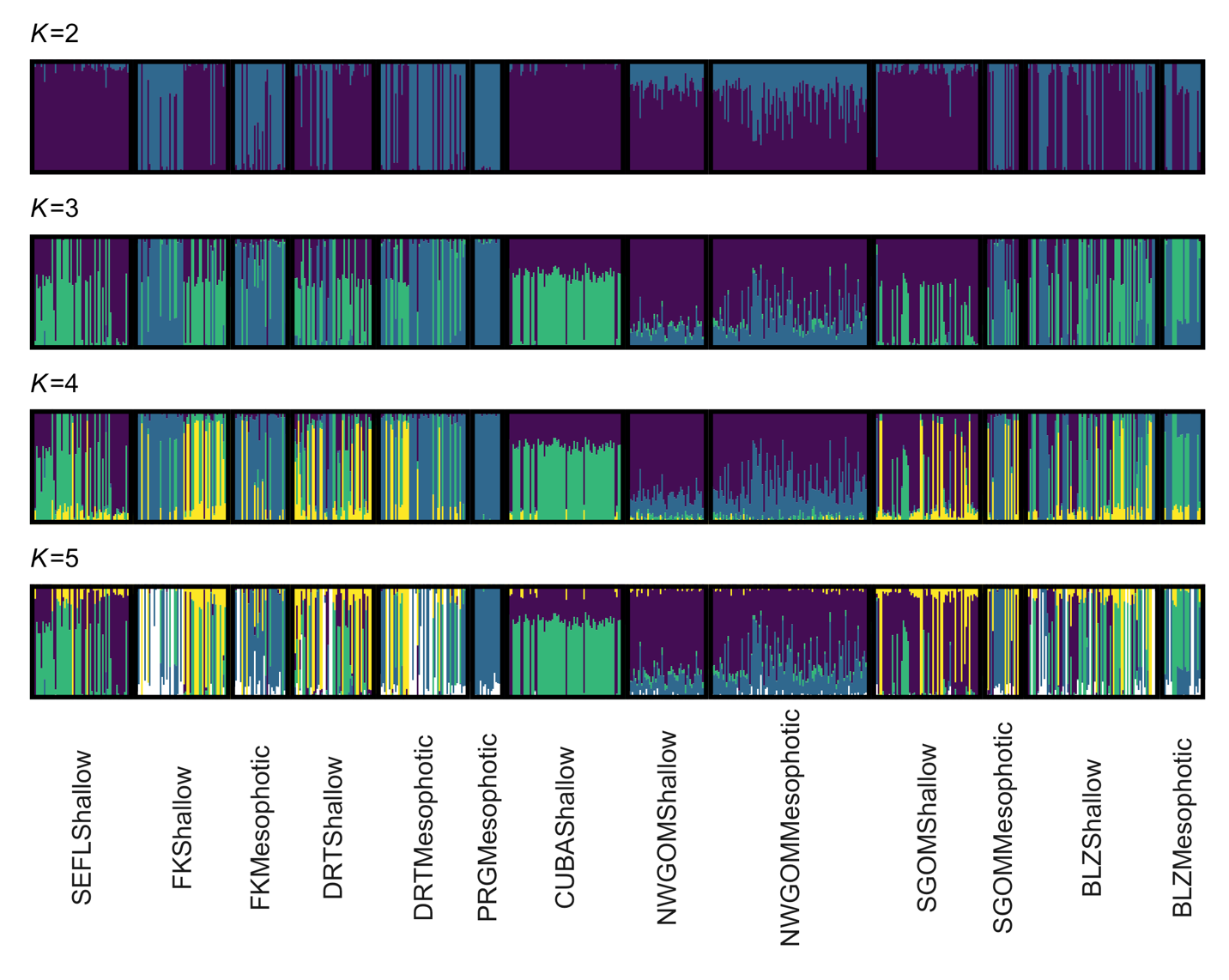
**

**Figure S1.** Admixture plots for *K* = 2 – 5 genetic clusters, represented by the colors purple, blue, green, yellow, and white. Each bar indicates individual *M. cavernosa* samples and the relative proportion of the colors represents the relative likelihood of membership to each of the proposed genetic clusters. The mesophotic Cuba population is not displayed because there were only two sample replicates, but they remained dominated by the blue genetic cluster across all values of *K.* All samples are ordered by geographic sampling site, except for Pulley Ridge which only had one sampling site. For Southeast Florida (SEFL), Florida Keys (FK), Dry Tortugas (DRT), and Belize (BLZ) samples are ordered from northernmost site to southernmost site. For Cuba, the Northwest Gulf of Mexico (NWGOM), and Southern Gulf of Mexico (SGOM) samples are ordered from westernmost site to easternmost site.
